# The Impact of Diet Wheat Source on the Onset of Type 1 Diabetes Mellitus—Lessons Learned from the Non-Obese Diabetic (NOD) Mouse Model

**DOI:** 10.3390/nu9050482

**Published:** 2017-05-10

**Authors:** Jonathan Gorelick, Ludmila Yarmolinsky, Arie Budovsky, Boris Khalfin, Joshua D. Klein, Yosi Pinchasov, Maxim A. Bushuev, Tatiana Rudchenko, Shimon Ben-Shabat

**Affiliations:** 1 Eastern Regional Research and Development Center, Judea Center, Kiryat Arba 90100, Israel; yludmila@post.bgu.ac.il (L.Y.); abudovsky@gmail.com (A.B.); 2 Department of Clinical Biochemistry and Pharmacology, Ben-Gurion University of the Negev, Beer Sheva 84990, Israel; boriskh83@gmail.com (B.K.); sbs@bgu.ac.il (S.B.-S.); 3 Institute of Plant Sciences, ARO-Volcani Center, Rishon LeZion 50250, Israel; vcjosh@volcani.agri.gov.il; 4 Siap Laboratory, Rehovot 76267, Israel; siap.013.lab@gmail.com; 5 Department of Information Science and Systems, Morgan State University, Baltimore, MD 21251, USA; maxim.bushuev@morgan.edu; 6 Scheller College of Business at Georgia Tech, Atlanta, GA 30308, USA; tatiana.rudchenko@scheller.gatech.edu

**Keywords:** type 1 diabetes mellitus, wheat, Non-Obese Diabetic (NOD) mouse, emmer, *Triticum dicoccoides*

## Abstract

Nutrition, especially wheat consumption, is a major factor involved in the onset of type 1 diabetes (T1D) and other autoimmune diseases such as celiac. While modern wheat cultivars possess similar gliadin proteins associated with the onset of celiac disease and T1D, alternative dietary wheat sources from Israeli landraces and native ancestral species may be lacking the epitopes linked with T1D, potentially reducing the incidence of T1D. The Non-Obese Diabetic (NOD) mouse model was used to monitor the effects of dietary wheat sources on the onset and development of T1D. The effects of modern wheat flour were compared with those from either *T. aestivum*, *T. turgidum* spp. *dicoccoides*, or *T. turgidum* spp. *dicoccum* landraces or a non-wheat diet. Animals which received wheat from local landraces or ancestral species such as emmer displayed a lower incidence of T1D and related complications compared to animals fed a modern wheat variety. This study is the first report of the diabetogenic properties of various dietary wheat sources and suggests that alternative dietary wheat sources may lack T1D linked epitopes, thus reducing the incidence of T1D.

## 1. Introduction

The incidence of Type 1 Diabetes (T1D), an autoimmune disease causing damage to the pancreatic beta cells, has doubled in the last decade [1]. The development of T1D involves a complex interaction between a person’s genetic background and environmental factors such as nutrition [2]. A significant increase of T1D in Sweden was associated with radical changes of food habits including increased consumption of pasta, white bread, meat, cheese, low-fat milk, exotic fruits, soda, and wheat-based snacks [3]. Adverse health effects of cereal proteins [2,4,5], cow’s milk proteins [6,7], and low vitamin D [4,7] on the onset of T1D have been demonstrated. Worldwide, wheat is the main staple food after maize and rice, and it is not surprising that a number of studies have suggested dietary wheat to be diabetogenic in humans [4]. The diabetogenic effects of wheat have also been demonstrated in Non-Obese Diabetic (NOD) mice [2,8,9]. Furthermore, an association between wheat gluten and celiac disease, and co-occurrence of both celiac disease and T1D have been documented [4,10,11,12,13,14]. People with celiac disease are at risk for diabetes [11,12,13,14,15,16,17,18,19,20].

Wheat contains several types of storage proteins such as gliadins and glutenins known as gluten, as well as globulins, triticins, and enzymes [21]. It is still unclear whether wheat gluten or other wheat storage proteins are responsible for the increased onset of T1D. Gluten-containing wheat stimulated the onset of T1D both in humans and NOD mice [2,4,22,23], but the opposite picture was observed with a diet enriched with purified gluten which protected NOD mice from T1D development [24]. Both in vivo and in vitro studies have demonstrated a direct effect of gluten on the immune system and on pancreatic beta cells [4].

All of the above-mentioned studies were performed using wheat from modern cultivars, which were developed in pursuit of agriculturally desirable traits associated with increased yield and uniformity of grain ripening. Unintentionally, modern wheat may have also been bred to increase the incidence of celiac and related diseases by including auto-immune epitopes [11]. However, not all wheat sources are genetically identical. There is a heterogeneity in the content of storage proteins for different wheat cultivars [21,25] and landraces [26]. In fact, the level of the celiac-related Glia-a9 epitope was much higher in modern wheat varieties than in the ancient landraces [21,25].

Unlike modern wheat cultivars, which possess little genetic variability, especially in storage proteins, local Israeli wheat landraces are very heterogeneous [27]. Importantly, many of the ancestral species of wheat are also found in Israel including Emmer (*Triticum diococcoides*)—the “Mother of Wheat”. Although lacking some of the agriculturally desirable traits of modern *T. aestivum* cultivars, emmer possesses a wealth of genetic variations, which could easily be transferred to the modern wheat. Some attempts have been made previously to incorporate the disease and drought resistance qualities of the emmer and native wheat forms into the modern varieties [28,29], but to the best of our knowledge the potentially non-diabetogenic properties of the native Israeli wheat landraces have not been studied.

With this in mind, we have collected Israeli landraces of wheat from the Israel Gene Bank (ARO), and the Germplasm Bank of the Institute for Cereal Crops Improvement (ICCI) at Tel Aviv University including accessions of Emmer, *T. turgidum* ssp. *dicoccoides*, originating from various locations in Israel as well as local landraces of *T. aestivum*. The types of wheat selected were: a modern bread wheat cultivar (*T. aestivum)* as a control, and landraces of *T. aestivum, T. turgidum* ssp. *dicoccoides* and *dicoccum.* The chosen wheat cultivars were tested on the NOD mouse model to estimate the effects of various dietary wheat sources on the onset of T1D in these animals. The aim of this study was to identify a low diabetogenic diet in which ‘standard’ wheat proteins were replaced with alternatives from native Israeli wheat landraces. Our hypothesis was that alternative dietary wheat sources, lacking the epitopes linked with T1D, may reduce the incidence of T1D.

## 2. Materials and Methods

### 2.1. Animals

NOD female mice, aged six weeks, were imported from Jackson Laboratories (USA) and cared for according to the guidelines set forth by the Ben-Gurion University of the Negev Animal Care and Use Committee. Food and water were supplied ad libitum.

### 2.2. Wheat Varieties

Most of the wheat varieties used in this work were grown on a sandy soil in a screen house at the Agricultural Research Organization—Volcani Center in Bet Dagan, Israel. Seeds of landraces of *T. aestivum*, *T. turgidum* ssp. *dicoccoides*, and *T. turgidum* ssp. *dicoccum* obtained from the Israel Gene Bank were sown in mid-November after incorporation of nitrogen (in the form of urea pellets) into the soil. Plants subsisted mostly on rainwater, with supplemental irrigation as needed from overhead sprinklers. Seeds were harvested during the last week of May and the first two weeks of June, depending on the ripening of each variety. Some of the emmer wheat flour was ground using seeds that were bulk-stored under controlled conditions at the Institute for Cereal Crops Improvement at the University of Tel Aviv. Conventional bread wheat grains (*Triticum aestivum*) were obtained from a commercial flour mill. Harvested seeds were analyzed for nutritional content and were milled to produce the diets for the animal study.

### 2.3. Diets

Experimental diets (Table 1) were based on a standard diet known to be diabetogenic in NOD mice consisting of 8.4% meat hydrolysate, 6.5% soybean protein, 1% milk protein, 3.2% corn starch, 2.5% wheat, and 1% oat proteins [2]. For preparing the low diabetogenic diet, wheat protein was replaced with an alternative protein source so that the total protein content stayed unchanged. For other diets, standard wheat proteins were replaced with wheat proteins from *T. aestivum*, *T. turgidum* spp. *dicoccoides*, or *T. turgidum* spp. *dicoccum* landraces. All tested diets had similar nutritional content (Table S1) including fat, protein, minerals, and amino acids.

### 2.4. Experimental Design

After four weeks of adaptation, animals were randomly divided into five groups (diets) of 10 animals (five per cage), with food supplied ad libitum. Initial parameters including weight, body length and fasting glucose levels were measured. The mice were inspected for diabetes daily using test strips for rapid determination of glucose in urine (Machenery-Nagel, Duren, Germany). In addition, glucose blood testing was performed approximately every two weeks during the experiment. Mice were considered diabetic if they had a urine glucose value above 150 mg/dL and a fasting blood glucose level of over 130 mg/dL [30]. The experimental diets lasted for 72 days, after which blood insulin, glucose, and cholesterol levels were measured after overnight fasting. In addition, IFN-γ and IL-10 levels were measured [28] using ELISA kits (Wuhan EIAab Science, Wuhan, China) according to the manufacturer’s instructions. Standard curves were generated for each plate to determine sample concentration. Absorbance was determined using SpectraMax Paradigm multi-mode detection platform (Molecular Devices, Sunnyvale, CA, USA) and data were analyzed using GraphPad Prism software (version 6; GraphPad Software, La Jolla, CA, USA).

### 2.5. Statistical Analysis

Data were analyzed using IBM SPSS Statistics software. The differences in five diet groups for all four parameters were tested using one-way ANOVA with α-level of 1%. Box plots by groups were constructed in order to represent visually the analysis of results. Kaplan Meier survival analysis was performed.

## 3. Results

In the framework of this study, five diets were prepared: (1) a low diabetogenic (non-wheat) diet; (2) a standard diabetogenic wheat bread containing diet; (3) a *T. aestivum* landrace diet; (4) a *T. turgidum* ssp. *dicoccoides* landrace diet; and (5) a *T. turgidum* spp. *dicoccum* landrace diet (Table 1and Table S1). The overall mortality for all of the groups was very low with a cumulative probability of survival (Kaplan-Meier) of 0.975. Although all mice consumed approximately equal amounts of food regardless of diet (results not shown), gains in body mass and length were influenced by the diet (*p* < 0.001) (Results not shown). Consumption of the standard diabetogenic wheat bread containing Diet 2 led to the highest body mass followed by Diets 3 and 4. At the end of the experiment, NOD mice on Diet 2 were significantly heavier (12.07 ± 1.96 g) than animals on Diet 4 (8.4 ± 1.91 g), and Diet 5 (7.02 ± 0.48 g). Mean body mass (9.97 ± 1.69 g) of animals on Diet 1 was intermediate. Animals on Diets 3–5 had significantly longer bodies (15–20%) than animals that received either a non-wheat diet, or the standard bread wheat containing diet (results not shown).

**Figure 1:**
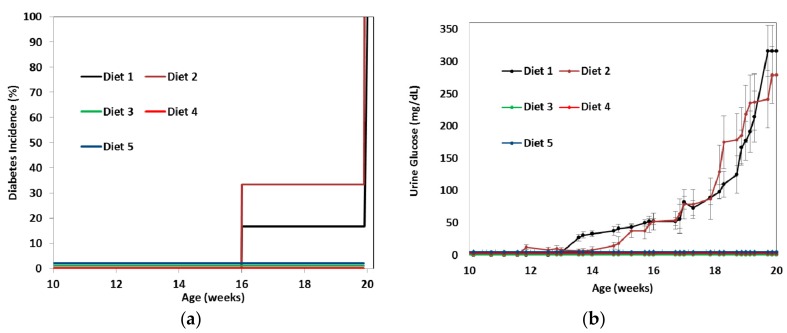

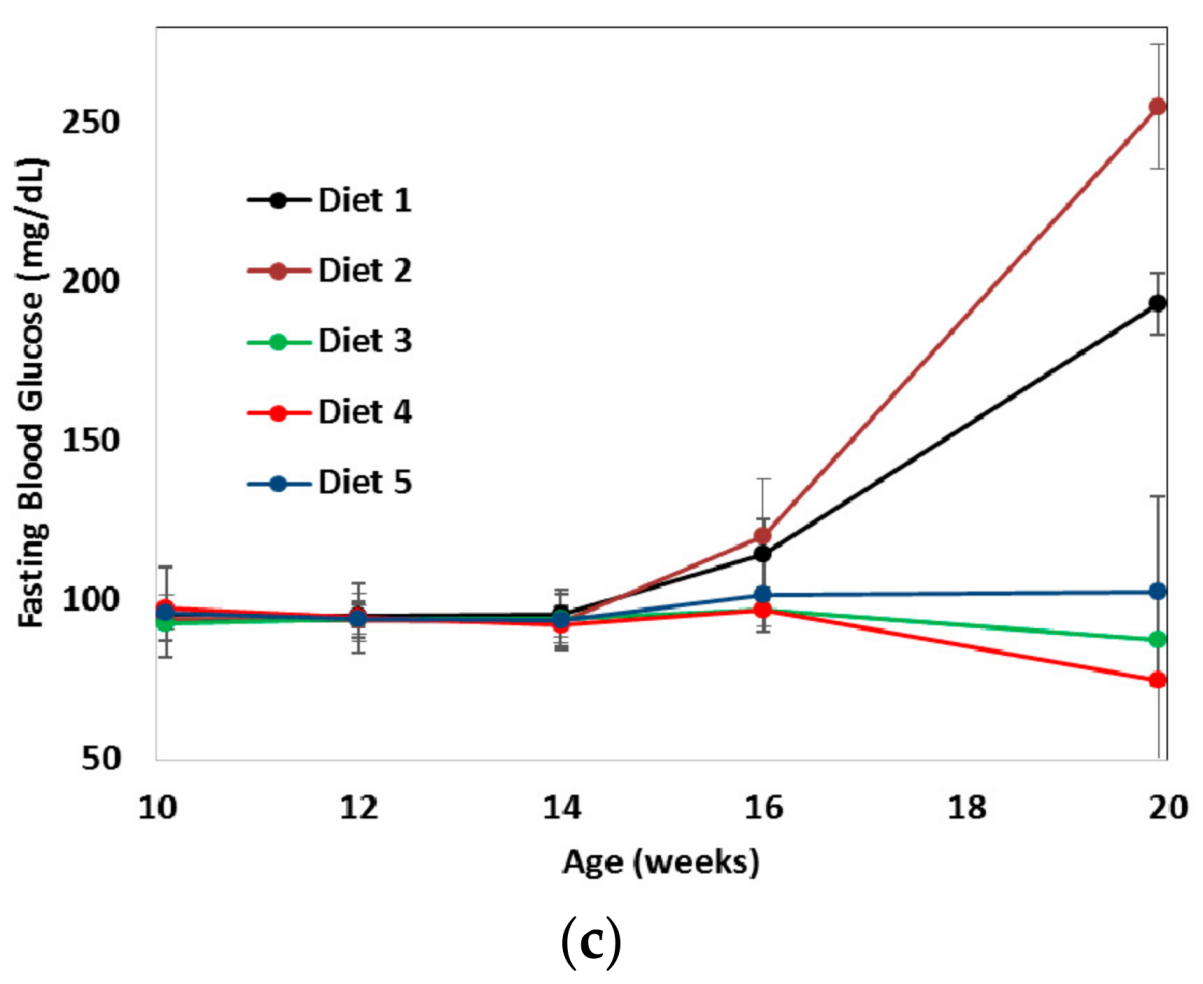
Onset of diabetes. Six week old female NOD mice received one of five different wheat diets for 14 weeks. The incidence of diabetes (**a**) was determined based on urine glucose (**b**) and fasting blood glucose levels (**c**). Data expressed in (**b**) and (**c**) represent the mean ± SE and (a) is the percentage of mice with elevated urine (>150 mg/dL) and fasting blood glucose levels (>130 mg/dL). Diets given: (1) low diabetogenic (non-wheat), (2) standard diabetogenic wheat bread, (3) *T. aestivum* landrace, (4) *T. turgidum* ssp. *dicoccoides* landrace, and (5) *T. turgidum* spp. *dicoccum* landrace.

The onset of diabetes was observed only in animals on Diets 1 and 2 (Figure 1). The incidence of diabetes (Figure 1a) in mice was determined according to the following criteria: elevated urine (>150 mg/dL; Figure 1b) and fasting blood glucose levels (>130 mg/dL; Figure 1c).

**Figure 2:**
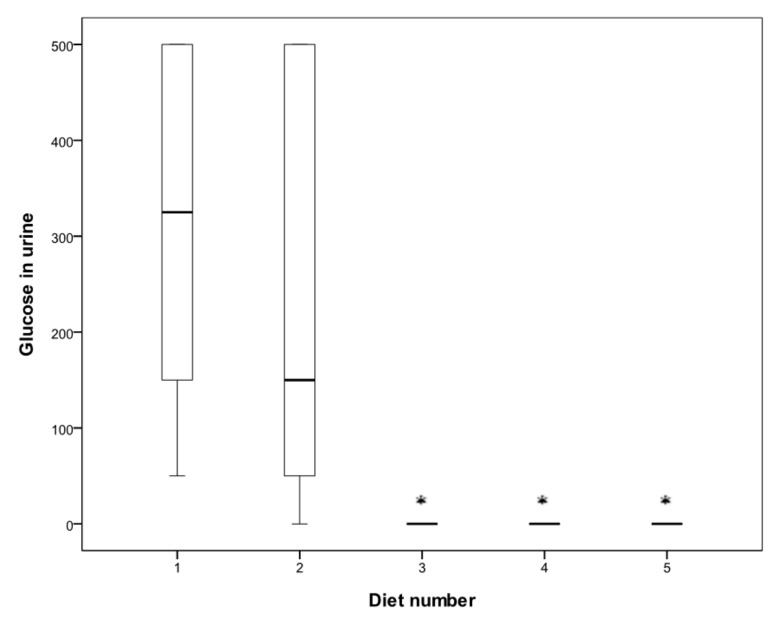
Glucose levels (mg/dL) in urine after 72 days. Diets given: (1) low diabetogenic (non-wheat), (2) standard diabetogenic wheat bread, (3) *T. aestivum* landrace, (4) *T. turgidum* ssp. *dicoccoides* landrace, and (5) *T. turgidum* spp. *dicoccum* landrace.

For the first six weeks of the study, there was no difference in fasting blood glucose levels between the animals on different diets (Figure 1c). By day 72, when the mice were 20 weeks old, there was already a significant difference in the glucose blood levels between the diet groups (*p* < 0.001). Glucose in urine was not detectable in animals on Diets 3, 4 and 5, even at the end of the experiment, while animals on Diets 1 and 2 had significantly elevated glucose in urine (Figure 1b and Figure 2).

**Figure 3:**
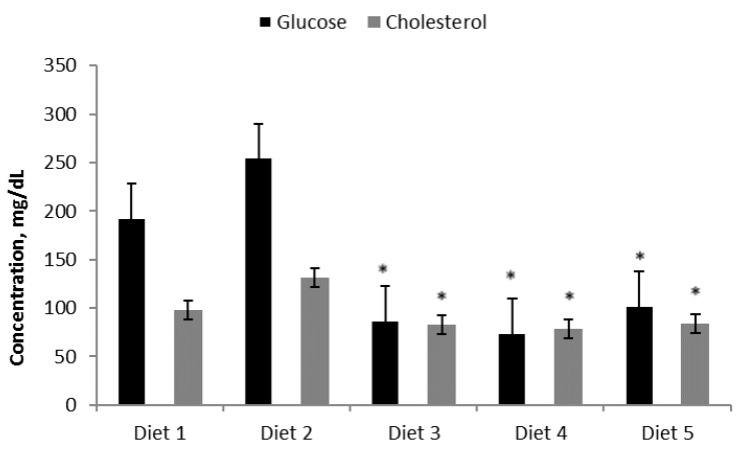
Fasting blood glucose (mg/dL) and cholesterol (mg/dL) levels after 72 days. Diets given: (1) low diabetogenic (non-wheat), (2) standard diabetogenic wheat bread, (3) *T. aestivum* landrace, (4) *T. turgidum* ssp. *dicoccoides* landrace, and (5) *T. turgidum* spp. *dicoccum* landrace. Results are presented as means ± standard errors. Differences between Diets 1 and 2 and Diets 3–5 were significant (*p* < 0.001).

**Figure 4:**
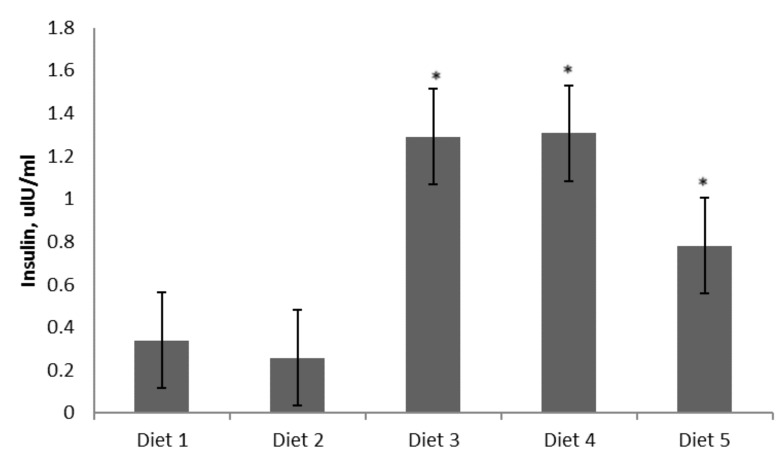
Fasting blood insulin (uIU/mL) levels after 72 days. Diets given: (1) low diabetogenic (non-wheat), (2) standard diabetogenic wheat bread, (3) *T. aestivum* landrace, (4) *T. turgidum* ssp. *dicoccoides* landrace, and (5) *T. turgidum* spp. *dicoccum* landrace. Results are presented as means ± standard errors. Differences between Diets 1 and 2 and Diets 3–5 were significant (*p* < 0.001).

After 72 days, fasting blood glucose levels increased in animals fed Diets 1 (193.2 ± 21.9 mg/dL) and 2 (255.0 ± 24.5 mg/day), compared to animals fed Diets 3 (87.4 ± 13.45 mg/day), 4 (74.6 ± 16.3 mg/day), and 5 (102.7 ± 21.2 mg/day) (Figure 3). The highest cholesterol level was observed for animals fed Diet 2 (Figure 3). With regard to the insulin level (measured in blood after overnight fasting), NOD mice on Diets 1 and 2 had the lowest insulin values (Figure 4), and differences between Diets 1 and 2 and Diets 3–5 were significant (*p* < 0.001). The fasting blood glucose:insulin ratios were over 10 times greater in Diet 2 than in Diets 3–4 and 6 times greater than in Diet 5.

**Figure 5:**
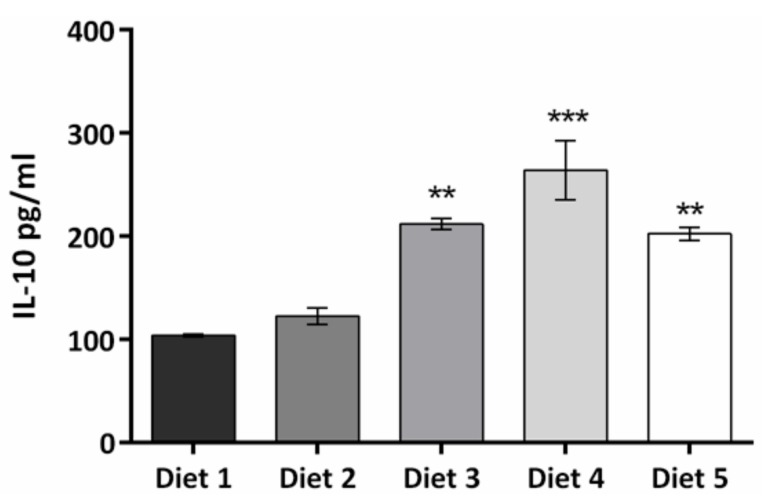
Anti-inflammatory cytokine IL-10 levels (pg/mL) from blood collected on day 72. Results are presented as means ± standard errors. Differences between Diets 1 and 2 and Diets 3–5 were significant (*p* < 0.01).

**Figure 6:**
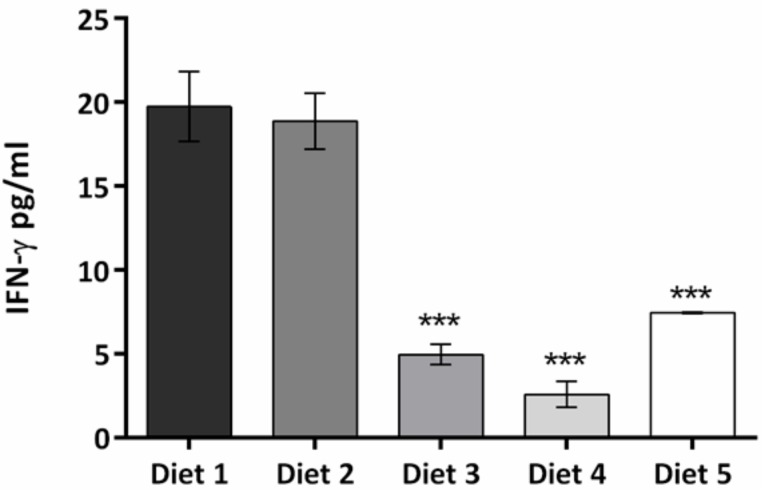
Pro-inflammatory cytokine IFN-γ levels (pg/mL) from blood collected on day 72. Results are presented as means ± standard errors. Differences between Diets 1 and 2 and Diets 3–5 were significant (*p* < 0.001).

Increased levels of the anti-inflammatory cytokine, IL-10, were observed in animals fed Diets 3–5 (*p* < 0.01 vs. control) (Figure 5), with the highest levels present in animals fed Diet 4. In contrast, levels of the pro-inflammatory cytokine, IFN-γ, were significantly reduced in Diets 3–5 (*p* < 0.001 vs. control) (Figure 6) with their IL-10:IFN-γ ratios 7, 15, and 4 times greater than Diets 1 and 2.

## 4. Discussion

This study is the first report of the diabetogenic properties of various ancestral dietary wheat sources. While in general wheat consumption has been linked to T1D [2,4,22,23], to the best of our knowledge there has never been an analysis of the effect of specific varieties of wheat on diabetogenesis. The NOD model mice are expected to develop T1D within 8–12 weeks after exposure to the standard diet [30]. Remarkably, animals on Diets 3, 4, and 5 did not have T1D for at least 72 days after initiation of the experiment. Diets based on the wheat progenitors *T. turdigum* spp. *dicoccoides* and *dicoccum* (Diets 4 and 5, respectively) were less diabetogenic than the modern conventional *T. aestivum* wheat cultivar (Diet 2). Surprisingly, a diet based on a landrace of *T. aestivum* (Diet 3), presumably of older lineage than the modern variety, was also less diabetogenic than the modern *T. aestivum*. Interestingly, reduced diabetogenicity correlated with reduced levels of IFN-γ, a proinflammatory cytokine involved in the autoimmune pathogenesis of T1D [31] as well increased levels of IL-10, an anti-inflammatory cytokine potentially implicated in hindering T1D development [32]. Somewhat unexpectedly, the non-wheat diet (Diet 1) displayed significant diabetogenic activity. Although this observation seems to contradict prior work, it is possible that the maize prolamins may also possess diabetogenic activity [33]. Overall, our results support the hypothesis that alternative dietary wheat sources may lack T1D linked epitopes, thus reducing the incidence of T1D.

Wheat flour contains a complex mixture of several types of storage proteins such as gliadins, glutenins, globulins, and triticins, [21]. Among them, the α-gliadins possess the most celiac-related toxic epitopes [21]. However, there may be other wheat proteins involved in the onset of T1D. For example, a wheat globulin protein Glb1 was identified as a diabetic related autoantigen using serum from diabetic BioBreeding rats [34]. Antibodies to this protein were also found in the serum of diabetic patients [35]. The proteins from the less diabetogenic wheat sources (*T. aestivum* landrace, *T. turgidum* spp. *dicoccoides*, or *T. turgidum* spp. *dicoccum*) have yet to be analyzed in order to unravel the mechanisms standing behind their reduced diabetogenicity. Future studies should address differences in expression of proteins capable of inducing autoimmune responses such as Glb1. Full characterization of the total protein makeup of various wheat sources will facilitate linking diabetogenic activity to specific proteins. This will aid in developing new less harmful wheat varieties which could greatly benefit public health, potentially reducing the incidence of diabetes and related complications.

## Figures and Tables

**Table 1:** Composition of the tested diets.

Diets Composition	Diet 1	Diet 2	Diet 3	Diet 4	Diet 5
Maize	35.00%	21.06%	21.06%	27.85%	21.06%
Wheat	0	20.00%	20.00%	20.00%	20.00%
Poultry Meat	15.15%	13.01%	13.01%	10.00%	13.01%
Corn Gluten Meal	5.27%	5.17%	5.17%	4.19%	5.17%
Sorghum	15.18%	14.92%	14.92%	11.63%	14.92%
Soya Oil	1.62%	1.00%	1.00%	1.46%	1.00%
Sunflower Meal	15.00%	15.00%	15.00%	15.00%	15.00%
Tomato Fibers	10.00%	6.84%	6.84%	6.68%	6.84%
ALIMET	0.04%	0.04%	0.04%	0.03%	0.04%
L-Lys-Cl	0.40%	0.47%	0.47%	0.50%	0.47%
NaCl	0.25%	0.27%	0.27%	0.29%	0.27%
Vit. Conc. BR	0.25%	0.25%	0.25%	0.25%	0.25%
Limestone	1.20%	1.24%	1.24%	1.26%	1.24%
Na_2_SO_4_	0.10%	0.10%	0.10%	0.10%	0.10%
Dicalcium Phosphate	0.54%	0.62%	0.62%	0.75%	0.62%
	100%	100%	100%	100%	100%

## Supplementary Materials

The following is available online at www.mdpi.com/ 2072-6643/9/5/482/s1, Table S1: Nutritional content of the tested diets.
