# Supplementary material for: The Impact of Diet Wheat Source on the Onset of Type 1 Diabetes Mellitus—Lessons Learned from the Non-Obese Diabetic (NOD) Mouse Model
Source: Nutrients. 2017 May 10;9(5):482. doi: 10.3390/nu9050482 (PMC5452212; doi:10.3390/nu9050482)
Supplement: Supplementary file 1 [file nutrients-09-00482-s001.pdf]

nutrients

MDPI

**Supplementary**

After preparation of the experimental diets, their nutritional content was analyzed in order to confirm comparable nutrient levels in all of the tested diets.

**Table S1.** Nutritional content of the tested diets.

| | Diet 1 | Diet 2 | Diet 3 | Diet 4 | Diet 5 |
|---|---|---|---|---|---|
| CRUDE PROT (%) | 23.7 | 23.6 | 23.6 | 23.6 | 23.6 |
| FAT (%) | 7.0 | 5.8 | 5.8 | 5.7 | 5.8 |
| ASH (%) | 5.3 | 5.4 | 5.4 | 5.3 | 5.4 |
| CRUDE FIBER (%) | 6.9 | 6.2 | 6.2 | 6.3 | 6.2 |
| CELLULOSE (%) | 4.1 | 4.5 | 4.5 | 4.5 | 4.5 |
| HEMI-CELLULOSE (%) | 5.4 | 6.3 | 6.3 | 6.4 | 6.3 |
| LIGNIN (%) | 2.9 | 2.7 | 2.7 | 2.6 | 2.7 |
| ADF (%) | 8.8 | 8.1 | 8.1 | 8.0 | 8.1 |
| NDF (%) | 15.3 | 14.8 | 14.8 | 14.9 | 14.8 |
| NON-N-EXTRACT (%) | 43.2 | 45.8 | 45.8 | 48.0 | 45.8 |
| SUGAR (%) | 1.5 | 1.9 | 1.9 | 1.9 | 1.9 |
| STARCH ac hydr (%) | 32.2 | 35.0 | 35.0 | 36.9 | 35.0 |
| CALCIUM (%) | 1.00 | 1.00 | 1.00 | 1.00 | 1.00 |
| PHOSPHORUS (%) | 0.63 | 0.63 | 0.63 | 0.63 | 0.63 |
| PHOSPHORUS av (%) | 0.35 | 0.35 | 0.35 | 0.35 | 0.35 |
| CHLORINE (%) | 0.30 | 0.31 | 0.31 | 0.31 | 0.31 |
| MAGNESIUM (%) | 0.19 | 0.19 | 0.19 | 0.19 | 0.19 |
| POTASSIUM (%) | 0.80 | 0.73 | 0.73 | 0.71 | 0.73 |
| SODIUM (%) | 0.28 | 0.28 | 0.28 | 0.28 | 0.28 |
| SULPHUR (%) | 0.18 | 0.19 | 0.19 | 0.19 | 0.19 |
| COPPER (mg) | 18.56 | 18.12 | 18.12 | 17.54 | 18.12 |
| IODINE (mg) | 1.26 | 1.27 | 1.27 | 1.26 | 1.27 |
| IRON (mg) | 108 | 110 | 110 | 108 | 110 |
| MANGANESE (mg) | 81 | 86 | 86 | 86 | 86 |
| SELENIUM (mg) | 0.60 | 0.59 | 0.59 | 0.57 | 0.59 |
| ZINC (mg) | 66 | 71 | 71 | 72 | 71 |
| NICKEL (mg) | 1.39 | 1.62 | 1.62 | 1.68 | 1.62 |
| FLUORINE (mg) | 0.67 | 0.92 | 0.92 | 0.93 | 0.92 |
| MOLYBDENUM (mg) | 0.39 | 0.40 | 0.40 | 0.38 | 0.40 |
| LYSINE (%) | 1.28 | 1.28 | 1.28 | 1.28 | 1.28 |
| METHIONINE (%) | 0.50 | 0.50 | 0.50 | 0.50 | 0.50 |
| CYSTEINE (%) | 0.46 | 0.48 | 0.48 | 0.48 | 0.48 |
| MET + CYS (%) | 0.93 | 0.95 | 0.95 | 0.96 | 0.95 |
| THREONINE (%) | 0.95 | 0.91 | 0.91 | 0.88 | 0.91 |
| TRYPTOPHAN (%) | 0.25 | 0.25 | 0.25 | 0.25 | 0.25 |
| ISOLEUCINE (%) | 1.06 | 1.04 | 1.04 | 1.01 | 1.04 |
| LEUCINE (%) | 2.05 | 1.96 | 1.96 | 1.96 | 1.96 |
| VALINE (%) | 1.16 | 1.15 | 1.15 | 1.16 | 1.15 |
| HISTIDINE (%) | 0.50 | 0.50 | 0.50 | 0.52 | 0.50 |
| ARGININE (%) | 1.47 | 1.43 | 1.43 | 1.41 | 1.43 |
| GLYCINE (%) | 1.30 | 1.24 | 1.24 | 1.20 | 1.24 |
| SERINE (%) | 1.14 | 1.12 | 1.12 | 1.12 | 1.12 |

| | | | | | |
|---|---|---|---|---|---|
| GLY + SER (%) | 2.41 | 2.34 | 2.34 | 2.29 | 2.34 |
| PHENYLALANINE (%) | 1.28 | 1.25 | 1.25 | 1.22 | 1.25 |
| TYROSINE (%) | 0.86 | 0.82 | 0.82 | 0.81 | 0.82 |
| PHE + TYROSINE (%) | 2.12 | 2.05 | 2.05 | 2.01 | 2.05 |
| ASPARTIC ACID (%) | 1.86 | 1.79 | 1.79 | 1.70 | 1.79 |
| GLUTAMIC ACID (%) | 3.56 | 3.96 | 3.96 | 4.30 | 3.96 |
| PROLINE (%) | 1.60 | 1.65 | 1.65 | 1.70 | 1.65 |
| ALANINE (%) | 1.40 | 1.34 | 1.34 | 1.29 | 1.34 |
